# Analysis of the Degradation of OCPs Contaminated Soil by the BC/nZVI Combined with Indigenous Microorganisms

**DOI:** 10.3390/ijerph20054314

**Published:** 2023-02-28

**Authors:** Qun Li, Lei Zhang, Jinzhong Wan, Tingting Fan, Shaopo Deng, Yan Zhou, Yue He

**Affiliations:** 1 Ministry of Ecology and Environment Peoples Republic of China, Nanjing Institute of Environmental Science, No. 8, Jiangwang Miao Street, Nanjing 210042, China; 2 State Environmental Protection Key Laboratory of Soil Environmental Management and Pollution Control, Nanjing 210042, China

**Keywords:** organochlorine pesticides, soil pollution, biochar-loaded nano-zero-valent iron, microbiological

## Abstract

Organochlorine pesticides (OCPs) were typical persistent organic pollutants that posed great hazards and high risks in soil. In this study, a peanut shell biochar-loaded nano zero-valent iron (BC/nZVI) material was prepared in combination with soil indigenous microorganisms to enhance the degradation of α-hexachlorocyclohexane(α-HCH) and γ-hexachlorocyclohexane(γ-HCH) in water and soil. The effects of BC/nZVI on indigenous microorganisms in soil were investigated based on the changes in redox potential and dehydrogenase activity in the soil. The results showed as follows: (1) The specific surface area of peanut shell biochar loaded with nano-zero-valent iron was large, and the nano-zero-valent iron particles were evenly distributed on the peanut shell biochar; (2) peanut shell BC/nZVI had a good degradation effect on α-HCH and γ-HCH in water, with degradation rates of 64.18% for α-HCH and 91.87% for γ-HCH in 24 h; (3) peanut shell BC/nZVI also had a good degradation effect on α-HCH and γ-HCH in soil, and the degradation rates of α-HCH and γ-HCH in the 1% BC/nZVI reached 55.2% and 85.4%, second only to 1% zero-valent iron. The degradation rate was the fastest from 0 to 7 days, while the soil oxidation-reduction potential (ORP) increased sharply. (4) The addition of BC/nZVI to the soil resulted in a significant increase in dehydrogenase activity, which further promoted the degradation of HCHs; the amount of HCHs degradation was significantly negatively correlated with dehydrogenase activity. This study provides a remediation strategy for HCH-contaminated sites, reducing the human health risk of HCHs in the soil while helping to improve the soil and increase the activity of soil microorganisms.

## 1. Introduction

Hexachlorocyclohexanes (HCHs), a group of persistent organochlorine pesticides (OCPs), were widely used in agriculture due to their long-term and effective insecticidal properties [1]. However, due to their toxicity and negative health effects, HCHs were listed as a persistent organic pollutant by the Stockholm Convention in 2009 and are now banned in most countries [2]. As a major agricultural country, China began producing HCHs in the 1960s and used 4.9 million tons, accounting for 33% of the world’s total consumption [3]. HCHs are stable in high temperatures, sunlight, and acidic conditions, and have a long half-life. In addition, the slow biotransformation between its isoforms leads to slow natural degradation, resulting in its persistence in the environment. Currently, HCHs residues can still be detected in soil in many areas of China. Yao et al. found agricultural soil contaminated with OCPs, such as HCH, in the Pearl River Delta [4]. Liu et al. found residues of HCHs and DDT in the sediment analysis of Xinhe and coastal wetlands in Yongding, with ecological risk assessments showing high risks [5]. Through sampling and analysis of sediment cores in the Yangzonghai Basin, Yuan et al. found the presence of organochlorine pesticide residues, primarily DDTs and HCHs [6]. Therefore, it is necessary to develop safe, economical, and feasible technical methods for decontaminating these contaminated sites.

Nanoscale zero-valent iron (nZVI) has the advantages of having a large specific surface area, strong reducibility, and environmental friendliness, making it a popular choice for the remediation of HCHs-contaminated water and soil in recent years [7,8]. However, there are still some limitations to the use of nZVI, such as its tendency to agglomerate and oxidize, incomplete dehalogenation, and poor migration, which can lead to low efficiency and potential ecological risks in the remediation of environmental pollution. Therefore, external modification methods are needed to address these issues. In order to overcome the limitations of nZVI, scholars have carried out loading modifications on nZVI [9,10,11], which have successfully avoided these defects. On the other hand, it can slow down the oxidation rate of nZVI and enhance its role in environmental remediation. In recent years, biochar (BC) has garnered significant attention from researchers in the fields of agriculture, resources, and the environment. This is due to its unique characteristics, such as a large specific surface area, a rich pore structure, and strong stability. As an excellent nZVI loading material, BC has become a research hotspot in the field of environmental remediation materials [12,13,14,15,16,17]. When used as a supporting material for nano-zero-valent iron, BC not only helps to overcome the defects of this material and reduce its oxidation rate, but also improves soil quality and increases the number and activity of soil microorganisms [18,19]. These properties make BC a promising material for addressing environmental challenges related to soil contamination and degradation. The degradation of OCPs via the reductive action of nZVI material results in the formation of benzene, which cannot be completely mineralized [20]. However, microorganisms have the ability to completely degrade and mineralize HCHs. Therefore, the use of microbial reduction is a promising method for the remediation of HCH-contaminated sites. The combination of nZVI and microbial remediation, known as nano-bioremediation technology, can achieve the complete degradation of HCHs [21,22,23].

However, the research on the degradation of HCHs by biochar/nano-zero-valent iron (BC/nZVI) materials at home and abroad has mainly focused on the aqueous phase system, and the degradation effect of HCHs in soil has rarely been reported [24,25]. In this study, peanut shell BC/nZVI, which was more conducive to the uniform adhesion of nZVI and maintains a large specific surface area after loading [24], was prepared and combined with soil indigenous microorganisms to enhance the degradation of HCHs (α-HCH and γ-HCH) in contaminated soil. The effect and mechanism of the synergistic degradation of soil HCHs by BC/nZVI in combination with indigenous microorganisms were investigated. This study provides a remediation strategy for HCH-contaminated sites, reducing the human health risk of HCHs in the soil while helping to improve the soil and increase the activity of soil microorganisms.

## 2. Methods

### 2.1. Soil Preparation

The test soil used in this study was collected from the site of a former agricultural pharmaceutical factory in Wuxi, China. The factory, which was established in 1956 and produced HCH powder, was closed and relocated in the late 1990s. The main pollutants in the soil were α-HCH and γ-HCH, with concentrations ranging from 10.737 to 11.574 mg/kg and 45.506 to 55.607 mg/kg, respectively. The soil particle size distribution was determined by the hydrometer method, and the test soil sample was a sandy loam according to the international soil texture classification standard. The basic physical and chemical properties of the soil were pH 7.96, moisture content 26.42%, and ORP 36.3 mV.

### 2.2. Preparation of BC/nZVI

A reducing agent was added uniformly and gradually into an aqueous solution of iron salt in the presence of a surfactant in order to prepare BC/nZVI [26,27]. As a result of the surfactant’s stabilizing and dispersing effects in the reaction system, the iron ions in the solution were reduced to singlet iron, which was then formed and loaded onto the BC. The preparation steps are as follows:

An amount of 5 g of FeSO_4_-7H_2_O was weighed and thoroughly dissolved in 500 mL of anhydrous ethanol/water (30:70, *v/v*) mixed solution. The solution was then transferred to a three-necked flask, to which the prepared BC was added at a ratio of 1:2 nZVI/BC (m/m) and thoroughly stirred with continuous nitrogen gas to remove dissolved oxygen from the solution. An amount of 1.71 g of NaBH_4_ was weighed and dissolved in 45 mL of deionized water to prepare a 1 mol/L reducing solution. Through a constant-pressure funnel, the reducing solution (NaBH_4_) was gradually introduced to the three-necked flask while stirring, with the drop rate of the solution being held constant at 1–2 drops per second. After the reduction reaction was finished, nitrogen kept being added until the reactor produced no detectable hydrogen (30 min). The reactive solution was then moved to a positive pressure filter. The filter residue (BC/nZVI) was then cleaned, dried at 70 degrees, and chilled. The entire process was completed in an N_2_ environment. The filter residue was then transferred to an anaerobic glove box with an N_2_ atmosphere, crushed into powder, and stored airtight.

### 2.3. Characterization of BC/nZVI

The surface area of the material was measured using a surface area analyzer (ASAP2010N, Mack Instruments, Atlanta, USA), and the surface topography of the sample was characterized using a scanning electron microscope (JSM-7600F, JEOL, Tokyo, Japan).

### 2.4. HCHs Degradation Experiments in Water and Soil

HCHs degradation experiments in water were performed using the batch balance method; for a detailed description of the procedure, Lin et al. can be referred to [24].

HCHs degradation experiments in soil were performed as follows [28]: The soil samples were loaded separately into 1 L black high-density polyethylene (HDPE) jars, each containing 500 g of soil sample. The sterilized experimental group, blank experimental group, 0.5% BC experimental group, 0.5% nZVI experimental group, 0.5% BC/nZVI experimental group, and 1% BC/nZVI experimental group were set up. Deionized water was added, and the soil samples were incubated at 25 °C for one week to recover soil microbial activity. Soil oxidation-reduction potential (ORP), dehydrogenase activity, and concentrations HCHs were sampled at 0, 7, 14, 21, 28, 35, 42, 56, 70, and 84 days for analysis. Each group was prepared in triplicate.

### 2.5. Detection of HCHs Concentration and Physicochemical Properties

The concentrations of α-HCH and γ-HCH in soil were measured using an ultrasonic method and detected by a gas chromatograph-mass spectrometer (6890N, Agilent Technologies, Santa Clara, USA). The extraction solution was n-hexane and acetone (analytically pure, Merk, Germany), and the detection temperature was 300 °C.

The soil ORP was determined using a potential method [29] and detected by redox electrodes (LE501, Mettler Toledo Instruments (Shanghai) Co., Shanghai, China).

The soil dehydrogenase activity was determined using the triphenyltetraazolyl chloride colorimetric method with the soil dehydrogenase kit (sDHA, Nanjing Jiancheng Bioengineering Institute, Nanjing, China) [30,31].

## 3. Results and Discussion

### 3.1. Results of Material Characterization

In the previous study by Lin et al. [24], the BC materials were screened from peanut shells, corn stalks, and straw. Finally, it was found that the peanut shell BC remained intact at a calcination temperature of 300 °C and did not collapse during the firing process. This was more conducive to the uniform adhesion of nZVI and maintained a large specific surface area after loading. The moderate porosity of the BC after firing was also beneficial for maintaining a large specific surface area [22].

**Figure 1:**
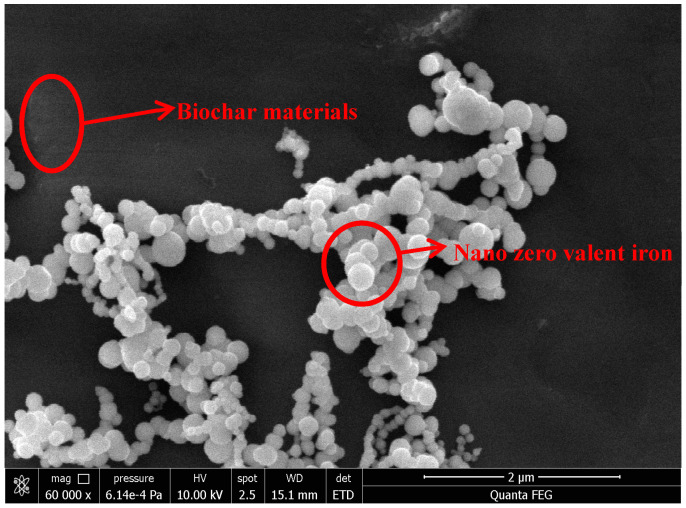
Scanning electron micrographs of different BC/nZVI.

In this study, the specific surface area analysis (BET) of nZVI, ZVI, and BC/nZVI is shown in Table 1. The specific surface area of the peanut shell BC/nZVI and nZVI was significantly higher than the 100 mesh ZVI powder. Scanning electron microscope (SEM) observations are shown in Figure 1. It can be seen that nZVI was evenly loaded on the surface of peanut shell BC without significant agglomeration. Due to the magnetic properties and attraction between the nZVI particles, the nZVI particles loaded on the surface of the BC present a chain structure [32].

### 3.2. Results of HCHs Degradation in Water

Lin et al. investigated the degradation effects of ZVI, nZVI, and the composite material (BC/nZVI) on HCHs in water and found that the composite material (BC/nZVI) had the best degradation effect [24]. This experiment verified the degradation performance of the composite material (BC/nZVI) for α-HCH and γ-HCH in an aqueous phase system, building upon the findings of previous studies.

**Figure 2:**
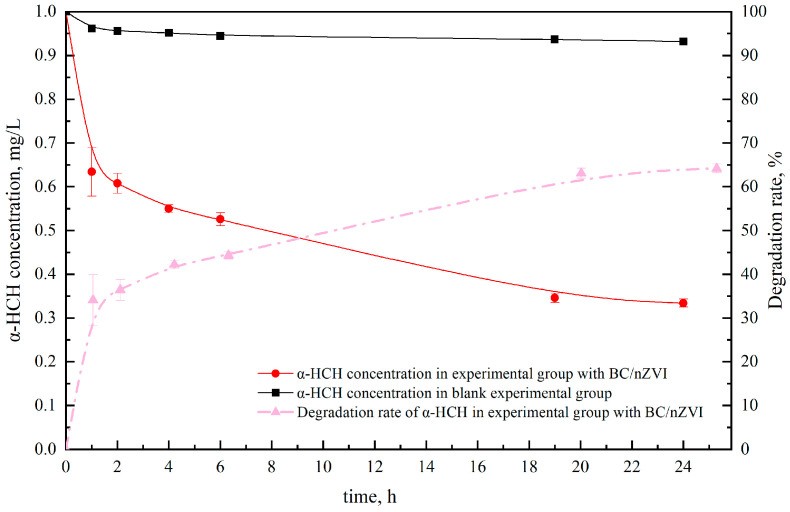
Effects of BC/nZVI material on the degradation of α-HCH in water.

**Figure 3:**
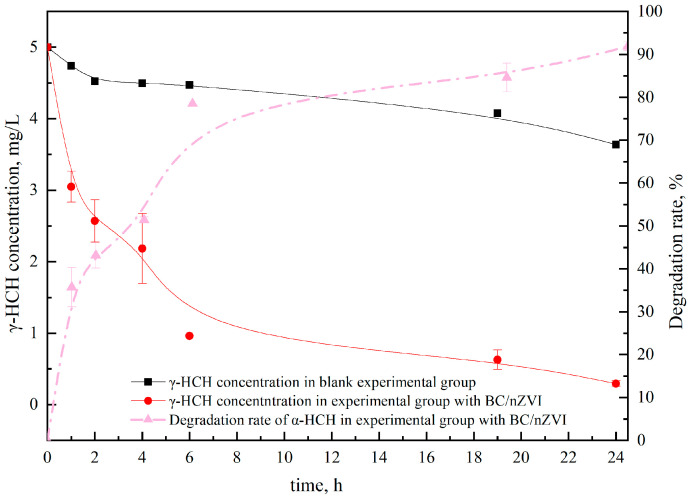
Effects of BC/nZVI material on the degradation of γ-HCH in water.

Figure 2 and Figure 3 show that the experimental group with the composite material had a significant degradation effect within 24 h. The residual concentration of α-HCH was 0.33 mg/kg within 24 h, with a degradation rate of 64.18%, and the residual concentration of γ-HCH was 0.30 mg/kg, with a degradation rate of 91.87%. These results demonstrate that the composite material has excellent potential for degrading α-HCH and γ-HCH and provide theoretical support for the subsequent degradation of HCH in soil. The degradation efficiency of α-HCH and γ-HCH was highest in the 0–1 h and 0–6 h periods, respectively. The degradation efficiency of α-HCH and γ-HCH is mainly due to the effect of nZVI. The difference in the spatial structure of α-HCH and γ-HCH isomers leads to the difference in the solubility of α-HCH and γ-HCH in the aqueous phase, resulting in a difference in the degradation rate in the aqueous phase system. The experimental results are in line with the findings of Sur S [33].

### 3.3. Results of HCHs Degradation in Soil and Its Effect on Microorganisms in Soil

#### 3.3.1. Results of HCHs Degradation in Soil

**Figure 4:**
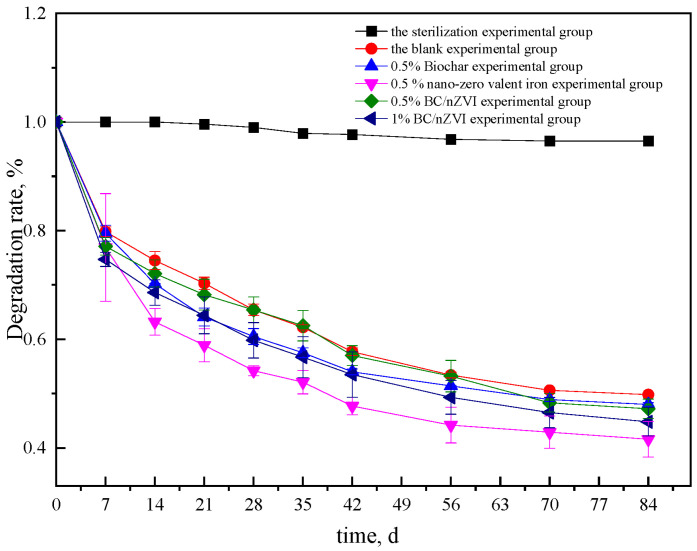
Effects of different experimental treatments on the degradation of α-HCH in soil.

**Figure 5:**
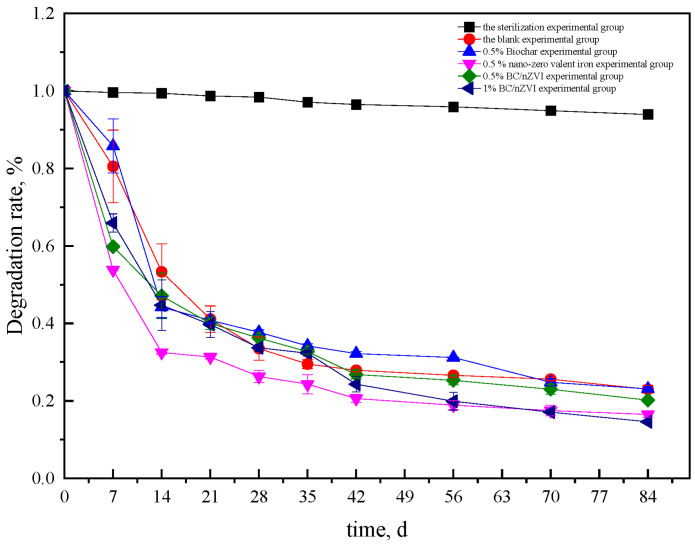
Effects of different experimental treatments on the degradation of γ-HCH.

Figure 4 and Figure 5 illustrate the degradation effects of different experimental groups on α-HCH and γ-HCH in soil. The concentrations of α-HCH and γ-HCH in the soil of the sterilization experimental group showed a slightly decreasing trend. The degradation rate of α-HCH was 3.5% with a degradation amount of 0.426 mg/kg after 84 days, while the degradation rate of γ-HCH was 6.1% with a degradation amount of 3.382 mg/kg, which may be mainly due to volatilization [34]. In the blank experimental group, the degradation rates of α-HCH and γ-HCH were 50.2% and 77.0% after 84 days, respectively, which may be due to the degradation effect of indigenous microorganisms. From day 0 to day 7, the degradation rate of α-HCH was 20.2%, but it slowed after that. The degradation rate of α-HCH remained stable from day 56 to day 84, between 0.8% and 2.8%. The degradation rate of γ-HCH decreased significantly from 0 to 14 days but slowed down after 14 days. The overall degradation trend was similar to that found by Meng Fanli [35]. It is possible that early microbial activity had just recovered and the overall demand for nutrients was greater, especially for microorganisms using HCHs as a carbon source, resulting in a high degradation rate in the early stage. However, due to the restriction of nutrients in the soil environment, microbial activity and abundance decreased in the later stage, leading to a slow decline in the degradation rate.

The concentration of α-HCH and γ-HCH in the soil of the BC experimental group decreased overall, which was similar to that of the blank experimental group. After 84 days, the degradation rate of α-HCH and γ-HCH in the BC group was 52%, 1.8% higher than that in the blank group. The overall degradation rate of γ-HCH was 76.9%, similar to that in the blank group. It should be noted that BC lacks the ability to degrade α-HCH. Instead, its effects are reflected in the promotion of the activity of degrading microorganisms, which leads to an improvement in the degradation rate of pollutants. Additionally, BC provides nutrients for microbial growth and reproduction, leading to an increase in the overall activity and abundance of microbial communities [36]. This finding was supported by the research of Samuel J. Gregory et al., who found that BC enhanced the degradation of HCHs by indigenous microorganisms in soils contaminated with arsenic and organochlorine pesticides [37].

The degradation effect of α-HCH was most effective in the soil at 0.5% nZVI, with a degradation rate of 58.4%, while the degradation rate of γ-HCH was 83.5%. In the first 14 days, the concentrations of both α-HCH and γ-HCH in the soil decreased significantly, likely due to the reduction and degradation of the nZVI. This finding is consistent with the research conducted by Yang et al., who observed a significant increase in the degradation rates of both α-HCH and γ-HCH in polluted soil after the addition of nZVI, roughly 10 days into the study [38].

The degradation rates of α-HCH and γ-HCH were evaluated in two groups of soil samples one containing 0.5% BC/nZVI and the other containing 1% BC/nZVI. The degradation of both α-HCH and γ-HCH was better in the 0.5% nZVI group compared to the 0.5% BC/nZVI group. After 84 days, the degradation rates of α-HCH and γ-HCH were 6% and 7.3% higher, respectively, in the 0.5% nZVI group compared to the 0.5% BC/nZVI group. One possible explanation for this was that the nZVI content of BC/nZVI was lower than that of single nZVI (the ratio of BC/nZVI to nZVI in the BC is 1:2, representing 0.5% of the total weight of the tested soil). As a result, the reduction of nZVI may play a more prominent role in the early rapid degradation, leading to a better degradation effect for the separate nZVI.

The degradation rate of α-HCH was similar to that of the 0.5% nZVI group when the amount of BC/nZVI was increased to 1%. However, for γ-HCH, the degradation effect of the 1% BC/nZVI group was superior to that of the 0.5% nZVI group. This demonstrates that the amount of added nZVI has a significant influence on the degradation effect. Interestingly, despite the lower content of nZVI in the 1% BC/nZVI group compared to the 0.5% nZVI group, the degradation effect was still improved. This suggests that the presence of BC may enhance the degradation effect of nZVI. There are several potential mechanisms for this improvement. Firstly, the porous structure and large surface area of BC provide a suitable habitat for microorganisms, allowing for effective adsorption and attachment of these organisms. The pores of BC also store water and nutrients necessary for microbial growth and reproduction. Secondly, the surface of BC contains easily decomposable carbon sources, nitrogen sources, and nutrient elements, providing favorable conditions for microbial growth and reproduction. Multiple studies have shown that both nZVI and BC have positive effects on soil microorganisms. BC can increase the number and activity of soil microorganisms, while nZVI can promote the microbial mineralization of organochlorine pollutants. As observed in the results of Chen Ting [39], the degradation effect of γ-HCH is generally better than that of α-HCH.

The results of ANOVA for the degradation rate of HCHs by different materials are shown in Table 2. When comparing BC and BC/nZVI, nZVI could significantly improve the rate of α-HCH degradation in soil. However, similar to the above results, both nZVI and BC/nZVI could significantly improve the degradation rate of γ-HCH in soil.

#### 3.3.2. The Effect of Material Addition on Soil Redox Potential

The ORP is a measure of the balance of oxidizing and reducing substances in the environment, which reflects the overall oxidizing or reducing properties of the system at a macroscopic scale. A lower ORP indicates stronger reducing properties of the environment, while a higher ORP indicates stronger oxidizing properties. Studies have demonstrated that soil environments with higher reducibility, or lower ORP values, are more conducive to the reduction and dechlorination of organic matter [40].

**Figure 6:**
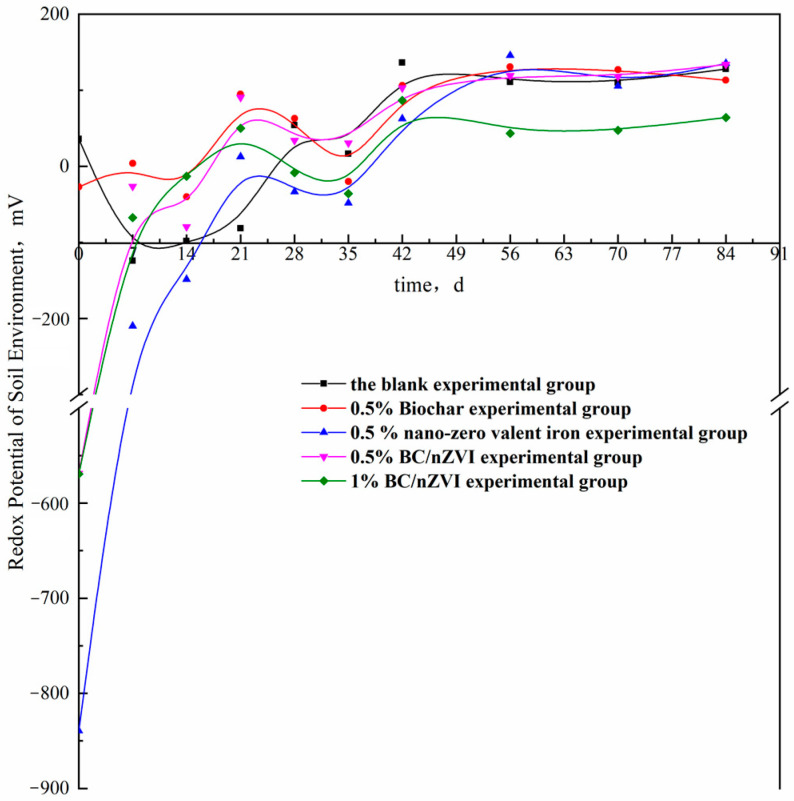
Effect of material addition on the redox potential of the soil environment.

Figure 6 demonstrates the effect of BC, nZVI, and BC/nZVI on soil redox potential. The trend of soil redox potential after adding 0.5% BC was similar to that of the blank group and fluctuated between −200 mV and 200 mV, indicating a weak reducing condition. However, after adding 0.5% nZVI, 0.5% BC/nZVI, and 1% BC/nZVI, the soil redox potential decreased sharply to −841.53 mV, −571.70 mV, and −573.47 mV, respectively. The entire soil environment showed strong reducibility, with the nZVI group showing the strongest reducibility (likely due to the higher concentration of nZVI in this group).

Over time, the redox potential of the three groups increased sharply within 0–14 days, eventually recovering to the range of −200 mV to 200 mV. As shown in Figure 4 and Figure 5, the concentrations of HCHs in the 0.5% nZVI, 0.5% BC/nZVI, and 1% BC/nZVI groups decreased rapidly within 0–14 days. The decrease was more rapid than the change in ORP values, likely because nZVI has a strong reduction capability that can quickly alter the overall soil redox conditions. However, because it is prone to oxidation, the soil undergoes a sharp reduction from strong reduction to weak reduction within 14 days, which can further validate the strong reducibility of nZVI as a reason for the sharp decline in HCH concentrations.

#### 3.3.3. The Effect of Material Addition on Soil Dehydrogenase Activity

Soil dehydrogenases are a type of redox enzyme that play an important intracellular role. They catalyze the dehydrogenation of organic matter, primarily through the secretion of microorganisms, and play a key role in the intermediate transformation and transfer of hydrogen. These enzymes can be used to reflect the redox ability of microorganisms in soil and to characterize the active microorganisms and their activity in the degradation of organic matter [30,31].

**Figure 7:**
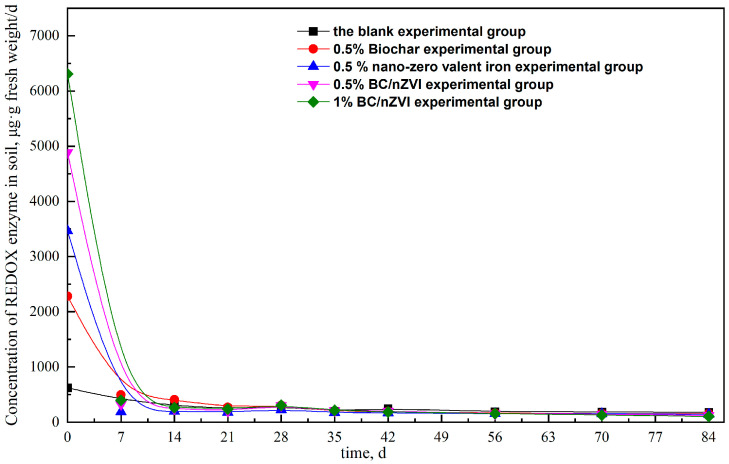
Effect of material addition on soil dehydrogenase activity.

According to Figure 7, the trend in the change of soil dehydrogenase after the addition of material was similar to that of the blank group, with a general trend of decreasing activity. The microbial biomass and microbial activity of secreted dehydrogenase reached their peaks at 0 days, with the peak values in the following order: 1% BC/nZVI > 0.5% BC/nZVI > 0.5% nZVI > 0.5% BC > blank. The activity of soil dehydrogenase in each experimental group decreased to less than 1000 μg·g fresh weight per day within 7 days of culture before stabilizing at this level.

The high activity of dehydrogenase observed in the experimental group during the early stages may be due to the recent addition of materials to the soil, which stimulated microorganisms to produce a large amount of dehydrogenase to facilitate the dechlorination of HCHs. This could explain the high degradation efficiency of HCHs during the early stages, as shown in Figure 4 and Figure 5 for the period of 0–7 days. These results were consistent with the previously observed trend of HCH degradation in Section 3.1, indicating that the addition of nZVI and BC can stimulate the secretion of dehydrogenase by microorganisms. The early stage of HCH degradation in each experimental group was found to be predominantly influenced by nZVI. In addition to stimulating the synergistic degradation of indigenous microorganisms, the synergistic effect of nZVI and BC was found to be stronger than the single effect.

## 4. Conclusions

In this study, batch experiments were performed to assess the possibility of BC/nZVI degradation on HCH-contaminated soil. The results showed that the specific surface area of peanut shell BC/nZVI was large, and the nano-zero-valent iron particles were evenly distributed on peanut shell biochar. The peanut shell BC/nZVI significantly improved the degradation rate of HCHs in soil, and the 1% BC/nZVI achieved 55.2% and 85.4% degradation of α-HCH and γ-HCH, respectively. The experimental group with the addition of nZVI exhibited stronger reducing properties, which caused a sharp increase in the redox potential in the soil from 0 to 14 d, while the concentrations of HCHs decreased rapidly. According to the changes of soil ORP, the strong reducing property of nZVI was one of the reasons for the degradation of α-HCH and γ-HCH in soil. Meanwhile, the addition of BC/nZVI to the soil resulted in a significant increase in dehydrogenase activity, indicating that BC/nZVI could enhance the activity of soil microorganisms. The dehydrogenase activity showed a significant correlation with the degradation of α-HCH and γ-HCH, indicating that the degradation of HCHs was related to the dehydrogenase activity, i.e., related to microorganisms.

Overall, the combination of BC/nZVI and indigenous microorganisms demonstrated effective removal of HCHs and provided a remediation strategy for HCH-contaminated sites. In the near future, BC/nZVI could be an appropriate material for reducing the human health risk of HCHs in the soil while helping to improve the soil and increase the activity of soil microorganisms. Based on the results of this study, the degradation scheme can be further optimized, such as adding specific strains of bacteria to further enhance the degradation of HCHs.

## Figures and Tables

**Table 1:** Composite material specific surface area of peanut shell biochar, nano zero-valent iron, and zero-valent iron.

Material Type	Specific Surface Area (M^2^/g)
Nano-zero-valent iron(nZVI)	34.2
Peanut shell BC/nZVI	30.31
Zero-valent iron (100 mesh)	0.9

**Table 2:** Multiple comparison of degradation rates on α-HCH and γ-HCH by different materials.

Matirials	a-HCH	P, 0.05 *	r-HCH	P, 0.05 *
sterilized	0.035125 ± 0.00	a	0.06082 ± 0.00	a
blank	0.502423 ± 0.00356	b	0.770248 ± 0.00985	b
0.5% BC	0.520494 ± 0.00319	bc	0.768934 ± 0.00512	b
0.5% nZVI	0.584231 ± 0.0325	c	0.835009 ± 0.00749	d
0.5% BC/nZVI	0.527969 ± 0.0178	bc	0.798349 ± 0.00134	c
1% BC/nZVI	0.551572 ± 0.0259	bc	0.854027 ± 0.00019	d

*: Same letters indicate non-significant difference; different letters indicate significant difference.

## Data Availability

Not applicable.
